# Synthesis of Ultrahigh Molecular Weight Polymers Containing Reactive Functionality with Low PDIs by Polymerizations of Long-Chain α-Olefins in the Presence of Their Nonconjugated Dienes by Cp*TiMe_2_(O-2,6-*^i^*Pr_2_C_6_H_3_)–Borate Catalyst

**DOI:** 10.3390/polym12010003

**Published:** 2019-12-18

**Authors:** Kotohiro Nomura, Sarntamon Pengoubol, Wannida Apisuk

**Affiliations:** Department of Chemistry, Tokyo Metropolitan University, 1-1 Minami Osawa, Hachioji, Tokyo 192-0397, Japan; sarntamon@gmail.com (S.P.); wannida.ppc@gmail.com (W.A.)

**Keywords:** polymerization, titanium complex, catalyst, α-olefin, nonconjugated diene, half-titanocene, borate, bottlebrush polymer

## Abstract

Copolymerizations of 1-decene (DC) with 1,9-decadiene (DCD), 1-dodecene (DD) with 1,11-dodecadiene (DDD), and 1-tetradecene (TD) with 1,13-tetradecadiene (TDD), using Cp*TiMe_2_(O-2,6-*^i^*Pr_2_C_6_H_3_) (**1**)–[Ph_3_C][B(C_6_F_5_)_4_] (borate) catalyst in the presence of Al*^i^*Bu_3_/Al(*n*-C_8_H_17_)_3_ proceeded in a quasi-living manner in *n*-hexane at −30 to −50 °C, affording ultrahigh molecular weight (UHMW) copolymers containing terminal olefinic double bonds in the side chain with rather low PDI (*M*_w_/*M*_n_) values. In the DC/DCD copolymerization, the resultant copolymer prepared at −40 °C possessed UHMW (*M*_n_ = 1.40 × 10^6^ after 45 min) with low PDI (*M*_w_/*M*_n_ = 1.39); both the activity and the PDI value decreased at low polymerization temperature (*M*_n_ = 5.38 × 10^5^, *M*_w_/*M*_n_ = 1.18, after 120 min at −50 °C). UHMW poly(TD-*co*-TDD) was also obtained in the copolymerization at −30 °C (*M*_n_ = 9.12 × 10^5^, *M*_w_/*M*_n_ = 1.51, after 120 min), using this catalyst.

## 1. Introduction

Transition metal catalyzed olefin polymerization is the core technology in the polyolefin industry, and the recent progress in the catalyst development provides new possibilities for the synthesis of new polymers [1,2,3,4,5,6,7,8,9,10,11,12,13,14]. Homopolymers of long-chain (higher) α-olefins are branched macromolecules with a high graft density, and the polymers are thus recognized as the simplest bottlebrush polymers, with their backbone and side chains consisting of alkanes [15,16]. Amorphous poly(α-olefin)s are used in hot-melt applications due to their high melt–flow rate with low density, and the ultrahigh molecular weight (UHMW) polymers possess highly entangled bottlebrush architectures and are used as drag-reducing agents (DRAs) in pipeline transport methods for crude oil and petroleum products for improvement of piping system capacity [17,18,19,20]. Recent reports revealed their melt structure, linear rheology, and interchain friction mechanism, including effect of side-chain length toward their linear viscoelastic response and melt microstructure [15,16].

However, reports for synthesis of UHMW polymers by polymerization of higher α-olefins (1-decene, 1-dodecene, 1-tetradecene, etc.) still have been limited [15,16,21,22], probably due to their preferred β-hydrogen elimination compared to the repeated insertion of monomer with a steric bulk (of alkane branching), as seen in ordinary metallocene catalysts yielding oligomers [21,23,24]. There are several examples for synthesis of (ultra)high molecular weight poly(1-hexene)s [21,25,26,27] by using [2,2-(O-4-Me-6-*^t^*Bu-C_6_H_3_)_2_S]TiCl_2_ (with water modified MMAO cocatalyst) [27,28], (C_5_HMe_4_)_2_HfCl_2_ (under ultrahigh pressure) [25], titanium complexes with diamine bis(phenolate) ligands [26]. Synthesis of rather high molecular weights poly(α-olefin)s, mostly poly(1-hexene)s, using the other catalysts, have also been known [24,29,30,31,32,33,34,35,36,37].

**Scheme 1:**
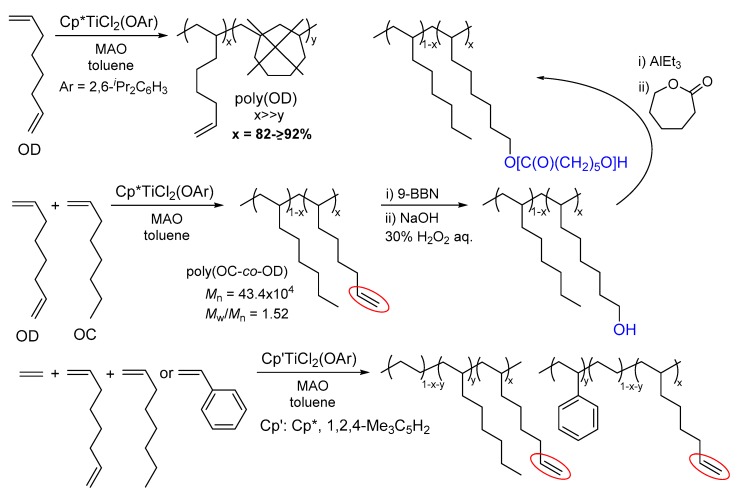
Polymerization of 1,7-octadiene (OD) and ethylene copolymerizations in the presence of OD, using Cp’TiCl_2_(O-2,6-*^i^*Pr_2_C_6_H_3_) (Cp’ = Cp*, 1,2,4-Me_3_C_5_H_2_)–MAO catalysts [38,44].

We reported that Cp*TiCl_2_(O-2,6-*^i^*Pr_2_C_6_H_3_)–MAO catalyst afforded high molecular weight poly(α-olefin)s by polymerizations of 1-decene (DC), 1-dodecene (DD), 1-hexadecene, and 1-octadecene [21]. It was then revealed that polymerizations of DC, DD, and 1-tetradecene (TD) proceeded in a quasi-living manner in the presence of Cp*TiMe_2_(O-2,6-*^i^*Pr_2_C_6_H_3_) (**1**)–[Ph_3_C][B(C_6_F_5_)_4_] (borate) catalyst and Al cocatalysts at −30 to −50 °C, affording UHMW polymers (e.g., poly(DC): *M*_n_ = 7.04 × 10^5^, *M*_w_/*M*_n_ = 1.37; poly(TD): *M*_n_ = 1.02 × 10^6^, *M*_w_/*M*_n_ = 1.38); the polymerizations proceeded with rather high catalytic activities (activity at −30 °C: 4120–5860 kg-poly(DC)/mol-Ti·h) [22]. The PDI (*M*_w_/*M*_n_) values decreased (accompanied with decrease in the catalytic activity) with an increase in the Al(*n*-C_8_H_17_)_3_/Al*^i^*Bu_3_ molar ratio and/or by decreasing the polymerization temperature (−40 and −50 °C). Moreover, it was demonstrated that 1,7-octadiene (OD) polymerization by Cp*TiCl_2_(O-2,6-*^i^*Pr_2_C_6_H_3_)–MAO catalyst afforded polymers containing terminal olefinic double bonds in the side chain without cyclization, cross-linking (Scheme 1). An introduction of polar functionality and the subsequent grafting (by living ring opening polymerization of ε-caprolactone) was also demonstrated into poly(1-octene-*co*-OD)s under mild conditions [38]. The method introduced a possibility for synthesis of functionalized polyolefins by incorporation of reactive functionalities, as demonstrated in the ethylene/1-octene or ethylene/styrene copolymerization in the presence of OD (synthesis of copolymers containing terminal olefinic double bonds with uniform compositions), and subsequent chemical modification under mild conditions (Scheme 1) [39,40,41,42,43,44,45].

**Scheme 2:**
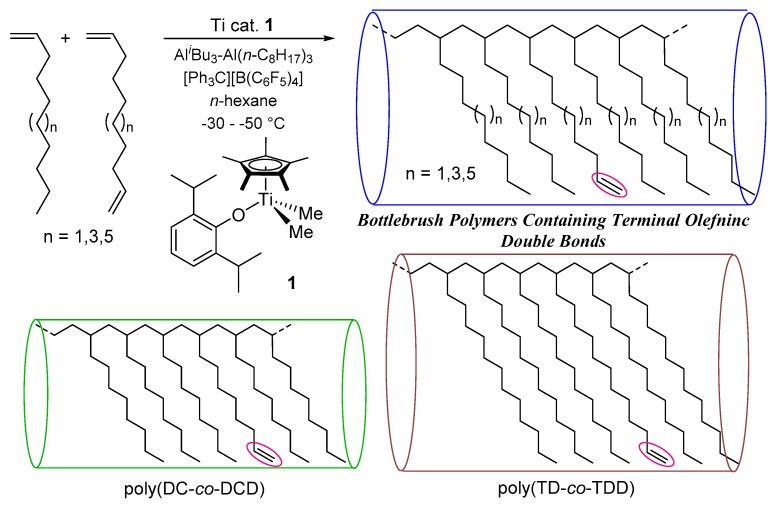
Copolymerization of 1-decene (DC), 1-dodecene (DD), 1-tetradecene (TD) with 1,9-decadiene (DCD), 1,11-dodecadiene (DDD), and with 1,13-tetradecadiene (TDD), using Cp*TiMe_2_(O-2,6-*^i^*Pr_2_C_6_H_3_) (**1**)–[Ph_3_C][B(C_6_F_5_)_4_] catalyst in the presence of Al cocatalyst.

Since, as described above, UHMW polymers are simple bottlebrush polymers prepared by polymerization of these higher α-olefins via the grafting-through approach, we thus have an interest in synthesis of the UHMW polymers containing terminal olefinic double bond by copolymerization of DC with 1,9-decadiene (DCD), DD with 1,11-dodecadiene (DDD), and TD with 1,13-tetradecadiene (TDD), using **1**–borate catalyst [46]. We thus, herein, wish to introduce our explored results for synthesis of new bottlebrush polymers with low PDIs containing reactive functionality in the side chain by **1**–borate catalyst in the presence of Al cocatalyst (Scheme 2).

## 2. Materials and Methods

All experiments were conducted in a dry box, under a nitrogen atmosphere, unless otherwise specified. All chemicals of reagent grade were purified by the standard purification protocols. The *n*-Hexane or toluene (anhydrous grade, Kanto Kagaku Co. Ltd., Tokyo, Japan) was stored in a bottle containing molecular sieves (mixture of 3A and 4A 1/16, and 13X) in the dry box, and was used without further purification. The 1-Decene, 1-dodecene, 1-tetradecene, 1,9-decadiene, 1.11-dodecadiene, and 1,13-tetradecadiene (reagent grades, TCI Co., Ltd., Tokyo, Japan) were stored in bottles, in the dry box, and were passed through an alumina short column prior to use. Cp*TiMe_2_(O-2,6-*^i^*Pr_2_C_6_H_3_) (**1**) was prepared according to our previous report [46], and [Ph_3_C][B(C_6_F_5_)_4_] (Asahi Glass Co. Ltd., Tokyo, Japan) was used as received.

All ^1^H and ^13^C NMR spectra were recorded on a Bruker AV 500 spectrometer (500.13 MHz for ^1^H; 125.77 MHz for ^13^C, Bruker Japan K.K., Tokyo, Japan) at 25 °C, and all chemical shifts in the spectra were recorded in ppm (reference SiMe_4_). Samples for the measurement were prepared by dissolving the polymers in 1,1,2,2-tetrachloroethane-*d*_2_ solution. Gel-permeation chromatography (GPC) were conducted for analysis of molecular weights (based on the calibration with standard polystyrene samples as the standard procedure) and the distributions. HPLC grade THF (degassed prior to use) was used for GPC analysis, and the GPC analysis was performed at 40 °C on a Shimadzu SCL-10A, using a RID-10A detector (Shimadzu Co., Ltd.), using degassed prior to use in THF (containing 0.03 wt.% of 2,6-di-*tert*-butyl-p-cresol, flow rate 1.0 mL/min). GPC columns (ShimPAC GPC-806, 804, and 802, 30 cm × 8.0 mm diameter, spherical porous gel made of styrene/divinylbenzene copolymer, ranging from <10^2^ to 2 × 10^7^ MW).

Typical polymerization procedures were as follows: in the dry box, 1-decene (30.0 mL), 1,9-decadiene (0.5 mL), *n*-hexane (30.0 mL), and Al*^i^*Bu_3_ and Al(*n*-C_8_H_17_)_3_ (prescribed amount) were added into a 100 mL round-bottom flask, which was connected to three-way valves. The flask was taken out from the dry box, and a toluene solution containing **1** (2.0 µmol/mL), which was pretreated with 2.0 eq. of Al*^i^*Bu_3_ at −30 °C, was then added into the mixture precooled at −30 °C under N_2_ atmosphere. The polymerization was started by the addition of a prescribed amount of toluene solution containing [Ph_3_C][B(C_6_F_5_)_4_] (2.0 µmol/mL). A certain amount (3.0 mL) of the reaction solution was taken out via a syringe from the reaction mixture, to monitor the time course; the sample solution was then quickly poured into *^i^*PrOH (150 mL) containing HCl (10 mL). The resultant polymer as precipitates was collected, adequately washed with *^i^*PrOH, and then dried in vacuo, for further analysis.

## 3. Results and Discussion

On the basis of our previous reports for polymerizations of 1-decene (DC), 1-dodecene (DD), and 1-tetradecene (TD) [22], and of 1,7-octadiene [38], Cp*TiMe_2_(OAr) (**1**, Ar = 2,6-*^i^*Pr_2_C_6_H_3_) was chosen as the catalyst precursor, and [Ph_3_C][B(C_6_F_5_)_4_] (borate) was chosen as the cocatalyst in the presence of Al*^i^*Bu_3_ and Al(*n*-C_8_H_17_)_3_ [22,29]. Copolymerizations of DC with 1,9-decadiene (DCD) were conducted in *n*-hexane at −30 to −50 °C, in the presence of Al cocatalyst [Al(*n*-C_8_H_17_)_3_/Al*^i^*Bu_3_/Ti = 400/100/1.0 (at −30 and −40 °C) or 300/200/1.0 (at −50 °C), molar ratio]; the ratios were used on the basis of the homo polymerization results [22]. As reported previously [22,29,47,48,49], use of Al(*n*-C_8_H_17_)_3_, weak reagent for alkylation, and/or chain transfer was effective to proceed without catalyst deactivation, probably not only due to a role as a scavenger, but also due to the fact that the Al alkyl would contribute to the stabilization of the catalytically active species by preventing the decomposition from further reaction with borate [50,51,52]. The results in the DC/DCD copolymerization are summarized in Table 1.

**Figure 1:**
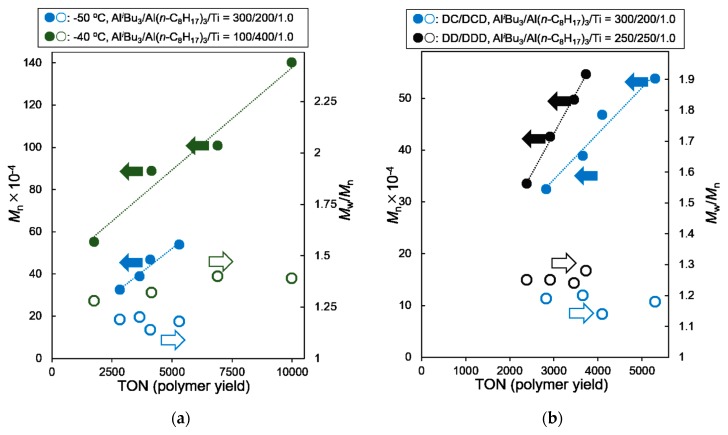
Plots of *M*_n_, *M*_w_/*M*_n_ *vs* polymer yields (turnover numbers, TON) in copolymerization of (**a**) 1-decene (DC) with 1,9-decadiene (DCD) and (**b**) 1-dodecene (DD) with 1,11-dodecadiene (DDD), using Cp*TiMe_2_(O-2,6-*^i^*Pr_2_C_6_H_3_) (**1**)–[Ph_3_C][B(C_6_F_5_)_4_] (borate) catalyst. (**b**) The results in DC/DCD copolymerization at −50 °C were plotted for comparison. Detailed data are shown in Table 1 and Table 2.

**Figure 2:**
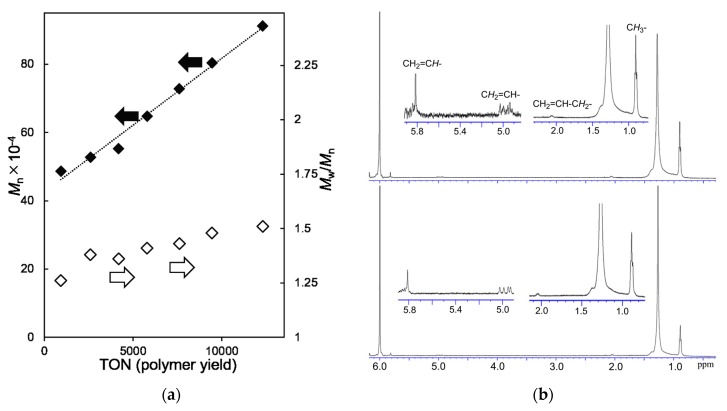
(**a**) Plots of *M*_n_, *M*_w_/*M*_n_ vs. polymer yields (turnover numbers, TON) in copolymerization of 1-tetradecene (TD) with 1,13-tetradecadiene (TDD), using Cp*TiMe_2_(O-2,6-*^i^*Pr_2_C_6_H_3_) (**1**)–borate catalyst. (**b**) Selected ^1^H NMR spectra (in 1,1,2,2-tetrachloroethane-*d*_2_ at 25 °C) for (top) poly(DC-*co*-DCD) (after five min) and (bottom) poly(TD-*co*-TDD) (after 60 min). The resonance at 5.8 ppm would be overlapped with the satellite of the resonance at 6.0 ppm.

As observed in the polymerization of DC, the copolymerization of DC with DCD proceeded with high catalytic activities (2750–7820 kg-polymer/mol-Ti·h within 20 min), even at −30 °C, affording high molecular weight polymers with rather narrow molecular weight distributions (run 1, *M*_n_ = 3.24 × 10^5^–7.53 × 10^5^, *M*_w_/*M*_n_ = 1.43–1.47). The *M*_n_ value increased over the time course, without significant changes in the PDI values. It turned out that the PDI values decreased at a low temperature, with a decrease in the catalytic activity; the resultant copolymer prepared at −40 °C possessed UHMW (run 2, *M*_n_ = 1.40 × 10^6^ after 45 min), with low PDI (*M*_w_/*M*_n_ = 1.39), and the PDI value became low when the copolymerization was conducted at −50 °C (run 3, *M*_n_ = 5.38 × 10^5^, *M*_w_/*M*_n_ = 1.18, after 120 min at −50 °C). As shown in Figure 1a, linear relationships between the *M*_n_ values and the polymer yields (turnover numbers, TON) were observed, suggesting that these polymerizations proceeded in a quasi-living manner, as reported in the polymerization of DC [22]. As shown in Figure 2b (shown below), the resultant copolymers contain terminal olefinic double bonds by incorporation of DCD. The content of DCD estimated by ^1^H NMR spectra slightly decreased gradually due to rather high consumption of DCD (rather high conversion of DCD and changes in the DCD concentration in the reaction solution) during the polymerization time course. This would suggest the possibility of (rather) gradient composition, although we do not have the firm elucidation at this moment.

Table 2 summarizes results in the DD/DDD copolymerization conducted at −40 and −50 °C. As observed in the DC/DCD copolymerization, the *M*_n_ value increased over the time course, without significant changes in the PDI values, and the PDI became low at −50 °C (run 5). The resultant copolymer prepared at −50 °C possessed high molecular weight with low PDI value (run 5, *M*_n_ = 5.46 × 10^5^, *M*_w_/*M*_n_ = 1.28 after 120 min). As shown in Figure 1b, a linear relationship between the *M*_n_ values and the polymer yields (turnover numbers, TON) was observed in the polymerization at −50 °C, suggesting a possibility of a quasi-living manner, as observed in the DC/DCD copolymerization.

Table 3 summarizes results in TD/TDD copolymerization conducted at −30 °C. Due to a difficulty of polymerization at low temperature (the *n*-hexane solution would be heterogeneous due to the freezing of TD), the polymerization could be conducted only at −30 °C, under rather diluted conditions. As observed in Table 1 and Table 2, the *M*_n_ value increased over the time course, without significant changes in the PDI values. The resultant copolymer possessed high molecular weight, with unimodal molecular weight distribution (run 6, *M*_n_ = 9.12 × 10^5^, *M*_w_/*M*_n_ = 1.51 after 120 min). As also shown in Figure 2a, a linear relationship between the *M*_n_ values and the polymer yields (turnover numbers, TON) was clearly observed. The results thus also suggest that the TD/TDD copolymerization proceeded in a quasi-living manner.

As shown in Figure 2b, the resultant polymers possessed a terminal olefinic double bond, as observed in poly(1-octene-*co*-1,7-octadiene) and poly(ethylene-*co*-1-octene-*co*-1,7-octadiene) [38], as well as in poly(ethylene-*co*-styrene-*co*-1,7-octadiene) [44] prepared by Cp*TiCl_2_(O-2,6-*^i^*Pr_2_C_6_H_3_)–MAO catalyst, and no resonances ascribed to protons in the internal olefins were observed (additional ^1^H NMR spectra are shown in the Supplementary Materials) [53]. The resultant polymers are highly soluble in toluene, THF, chloroform, dichloromethane, etc., without any difficulties (as seen in poly(1,5 hexadiene) containing partial cross-linking prepared by Cp_2_ZrCl_2_-MAO catalysts even under diluted conditions [54]). The results thus suggest that the resultant polymers were poly(DC-*co*-DCD)s and poly(TD-*co*-TDD)s containing terminal olefins in the side chain, as expected on the basis of our previous results [22,38].

## 4. Conclusions

We have shown that synthesis of ultrahigh molecular weight (UHMW) highly branched (bottlebrush) polymers that contain terminal olefinic double bonds in the side chain with rather low PDI (*M*_w_/*M*_n_) values has been attained by polymerization of long-chain (higher) α-olefins (1-decene (DC), 1-dodecene (DD), and 1-tetradecene (TD)) in the presence of corresponding nonconjugated dienes (1,9-decadiene (DCD), 1,11-dodecadiene (DDD), and 1,13-tetradecadiene (TDD), respectively), using Cp*TiMe_2_(O-2,6-*^i^*Pr_2_C_6_H_3_) (**1**)–[Ph_3_C][B(C_6_F_5_)_4_] (borate) as the catalyst, in the presence of Al*^i^*Bu_3_/Al(*n*-C_8_H_17_)_3_. These polymerizations proceeded in a quasi-living manner in *n*-hexane at −30 to −50 °C, and linear relationships between the *M*_n_ values and the polymer yields were observed in all cases, without significant changes in the PDI (*M*_w_/*M*_n_) values. The resultant poly(DC-*co*-DCD) prepared at −40 °C possessed UHMW (*M*_n_ = 1.40 × 10^6^ after 45 min) with low PDI (*M*_w_/*M*_n_ = 1.39), and UHMW poly(TD-*co*-TDD) was also obtained in the TD/TDD copolymerization at −30 °C (*M*_n_ = 9.12 × 10^5^, *M*_w_/*M*_n_ = 1.51, after 120 min). As described in the introduction, these polymers should possess highly branched bottlebrush architectures, and the present results strongly suggest a possibility of introduction of reactive functionality (terminal olefins) into the side chain (outside of the cylindrical structure). Moreover, as described in the introductory, as well as reported previously [38], an introduction of hydroxy group by treatment of the terminal olefinic double bonds with BBN and the subsequent grafting (by living ring opening polymerization of ε-caprolactone) would be possible. One issue we have not yet clarified clearly is the effect of diene monomers on the monomer reactivity ratio. We thus believe that the results could demonstrate providing new materials (functionalized polyolefin bottlebrush) based on polyolefins, and more details including further analysis and applications will be introduced in the future.

## Figures and Tables

**Table 1:** Copolymerization of 1-decene (DC) with 1,9-decadiene (DCD) by Cp*TiMe_2_(O-2,6-*^i^*Pr_2_C_6_H_3_) (**1**)–[Ph_3_C][B(C_6_F_5_)_4_] (borate) catalyst *^a^*.

Run	Al(*n*-C_8_H_17_)_3_/ Al*^i^*Bu_3_/Ti *^b^*	Temp. /°C	Time /min	Yield *^c^* /mg	Activity *^d^*	TON *^e^*	*M*_n_*^f^* × 10^−4^	*M*_w_/ *M*_n_*^f^*	DCD *^g^* /mol%	Conv. *^h^* /%
1	400/100/1.0	−30	5	652	7820	4650	32.4	1.43	8.9	16
			10	796	4780	5680	45.3	1.47	8.4	17
			15	864	3460	6160	52.4	1.45	8.2	19
			20	918	2750	6550	75.3	1.43	8.0	19
2	400/100/1.0	−40	10	246	1480	1760	55.1	1.28	9.1	6
			20	582	1750	4150	88.7	1.32	8.8	13
			30	966	1930	6900	100.7	1.40	7.8	19
			45	1400	1870	9990	140.1	1.39	5.6	20
3	300/200/1.0	−50	60	396	400	2830	32.4	1.19	9.8	10
			75	512	410	3660	38.9	1.20	9.2	12
			90	574	380	4100	46.8	1.14	8.3	13
			120	742	370	5300	53.8	1.18	7.4	15

*^a^* Conditions: **1** 1.0 µmol, 1-decene 30.0 mL, 1,9-decadiene 0.5 mL, *n*-hexane 30.0 mL, Al*^i^*Bu_3_/Al(*n*-C_8_H_17_)_3_/ [Ph_3_C][B(C_6_F_5_)_4_]/Ti = 100/400/3.0/1.0 molar ratio (200/300/1.0 at −50 °C), **1** was pretreated with 2.0 equiv of Al*^i^*Bu_3_ at −30 °C for 10 min before addition into the mixture. *^b^* Molar ratio. *^c^* A prescribed amount (3.0 mL) of the solution was removed via syringe from the reaction mixture, and the yields were based on obtained amount. *^d^* Activity in kg-polymer/mol-Ti·h. *^e^* TON (turnovers) = monomer consumed (mol)/mol-Ti. *^f^* GPC data in THF vs polystyrene standards. *^g^* Estimated by ^1^H NMR spectra. *^h^* Estimated ((DCD consumed/DCD charged) × 100) (conv. = conversion).

**Table 2:** Copolymerization of 1-dodecene (DD) with 1,11-dodecadiene (DDD) by Cp*TiMe_2_(O-2,6-*^i^*Pr_2_C_6_H_3_) (**1**)–[Ph_3_C][B(C_6_F_5_)_4_] (borate) catalyst *^a^*.

Run	Al(*n*-C_8_H_17_)_3_/ Al*^i^*Bu_3_/Ti *^b^*	Temp. /°C	Time /min	Yield *^c^* /mg	Activity *^d^*	TON *^e^*	*M*_n_*^f^* × 10^−4^	*M*_w_/ *M*_n_*^f^*	DDD *^g^* /mol%	Conv. *^h^* /%
4	400/100/1.0	−40	20	828	2480	5900	42.6	1.34	7.9	17
			30	897	1790	6390	48.3	1.39	7.7	18
			40	932	1400	6640	51.4	1.38	6.6	18
5	250/250/1.0	−50	75	402	320	2390	33.5	1.25		
			90	492	330	2920	42.6	1.25	7.7	10
			105	582	330	3460	49.7	1.24	7.5	11
			120	628	310	3730	54.6	1.28	7.5	12

*^a^* Conditions: **1** 1.0 µmol, 1-dodecene 25.0 mL, 1,11-dodecadiene 0.5 mL, *n*-hexane 35.0 mL, Al*^i^*Bu_3_/Al(*n*-C_8_H_17_)_3_/ [Ph_3_C][B(C_6_F_5_)_4_]/Ti = 100/400/3.0/1.0 molar ratio (250/250/1.0 at −50 °C), **1** was pretreated with 2.0 equiv of Al*^i^*Bu_3_ at −30 °C for 10 min before addition into the mixture. *^b^* Molar ratio. *^c^* A prescribed amount (3.0 mL) of the solution was removed via syringe from the reaction mixture, and the yields were based on obtained amount. *^d^* Activity in kg-polymer/mol-Ti·h. *^e^* TON (turnovers) = monomer consumed (mol)/mol-Ti. *^f^* GPC data in THF vs polystyrene standards. *^g^* Estimated by ^1^H NMR spectra. *^h^* Estimated ((DDD consumed/DDD charged) × 100).

**Table 3:** Copolymerization of 1-tetradecene (TD) with 1,13-tetradecadiene (TDD) by Cp*TiMe_2_(O-2,6-*^i^*Pr_2_C_6_H_3_) (**1**)–[Ph_3_C][B(C_6_F_5_)_4_] (borate) catalyst (−30 °C) *^a^*.

Run	Time/min	Yield *^b^*/mg	Activity *^c^*	TON *^d^*	*M*_n_*^e^* × 10^−4^	*M*_w_/*M*_n_*^e^*	TDD *^f^*/mol%
6	10	184	1100	927	48.7	1.26	
	30	516	1030	2600	52.8	1.38	4.5
	40	830	1110	4180	55.3	1.36	4.0
	60	1150	1150	5790	64.8	1.41	3.7
	75	1510	1210	7610	72.9	1.43	3.5
	90	1872	1250	9440	80.4	1.48	3.2
	120	2432	1220	12300	91.2	1.51	

*^a^* Conditions: **1** 1.0 µmol, 1-tetradecene 20.0 mL, 1,13-tetradecadiene 1.0 mL, *n*-hexane 40.0 mL, Al*^i^*Bu_3_/Al(*n*-C_8_H_17_)_3_/[Ph_3_C][B(C_6_F_5_)_4_]/Ti = 100/400/3.0/1.0, molar ratio, **1** was pretreated with 2.0 equiv of Al*^i^*Bu_3_ at −30 °C for 10 min before addition into the mixture. *^b^* A prescribed amount (3.0 mL) of the reaction mixture was removed via syringe from the polymerization mixture, and the yields were based on obtained amount. *^c^*Activity in kg-polymer/mol-Ti·h. *^d^* TON (turnovers) = monomer consumed (mol)/mol-Ti. *^e^* GPC data in THF vs polystyrene standards. *^f^* Estimated by ^1^H NMR spectra.

## Supplementary Materials

The following are available online at https://www.mdpi.com/2073-4360/12/1/3/s1. Figure S1: ^1^H NMR spectrum (in 1,1,2,2-tetrachloroethane-*d*_2_ at 25 °C) for poly(1-decene-*co*-1,9-decadiene) (run 1, after 5 min, 1,9-decadiene 8.9 mol%), Figure S2: ^1^H NMR spectrum (in 1,1,2,2-tetrachloroethane-*d*_2_ at 25 °C) for poly(1-decene-*co*-1,9-decadiene) (run 2, after 10 min, 1,9-decadiene 9.1 mol%), Figure S3: ^1^H NMR spectrum (in 1,1,2,2-tetrachloroethane-*d*_2_ at 25 °C) for poly(1-dodecene-*co*-1,11-dodecadiene) (run 4, after 30 min, 1,11-dodecadiene 7.7 mol%), Figure S4: ^1^H NMR spectrum (in 1,1,2,2-tetrachloroethane-*d*_2_ at 25 °C) for poly(1-dodecene-*co*-1,11-dodecadiene) (run 5, after 120 min, 1,11-dodecadiene 7.5 mol%), Figure S5: ^1^H NMR spectrum (in 1,1,2,2-tetrachloroethane-*d*_2_ at 25 °C) for poly(1-tetradecene-*co*-1,13-tetradecadiene) (run 6, after 30 min, 1,13-tetradecadiene 4.5 mol%), Figure S6: ^1^H NMR spectrum (in 1,1,2,2-tetrachloroethane-*d*_2_ at 25 °C) for poly(1-tetradecene-*co*-1,13-tetradecadiene) (run 6, after 60 min, 1,13-tetradecadiene 3.7 mol%).
