# Supplementary material for: Synthesis of Ultrahigh Molecular Weight Polymers Containing Reactive Functionality with Low PDIs by Polymerizations of Long-Chain α-Olefins in the Presence of Their Nonconjugated Dienes by Cp*TiMe2(O-2,6-iPr2C6H3)–Borate Catalyst
Source: Polymers (Basel). 2019 Dec 18;12(1):3. doi: 10.3390/polym12010003 (PMC7023581; doi:10.3390/polym12010003)
Supplement: Supplementary file 1 [file polymers-12-00003-s001.pdf]

Supplementary Materials

Synthesis of Ultrahigh Molecular Weight Polymers Containing Reactive Functionality with Low PDIs by Polymerizations of Long-chain α-Olefins in the Presence of their Nonconjugated Dienes by $Cp^*TiMe_2(O\text{-}2,6\text{-}^iPr_2C_6H_3)$–Borate Catalyst

Kotohiro Nomura*, Sarntamon Pengoubol and Wannida Apisuk

Department of Chemistry, Tokyo Metropolitan University, 1-1 Minami Osawa, Hachioji, Tokyo 192-0397, Japan; ktnomura@tmu.ac.jp

Contents

Selected ^{1}H NMR spectra (in 1,1,2,2-tetrachloroethane-d_2 at 25 ºC) for poly(1-decene-*co*-1,9-decadiene)s (Figures S1,2), poly(1-dodecene-*co*-1,11-dodecadiene)s (Figures S3,4), and poly(1-tetradecene-*co*-1,13-tetradecadiene)s (Figures S5,6).

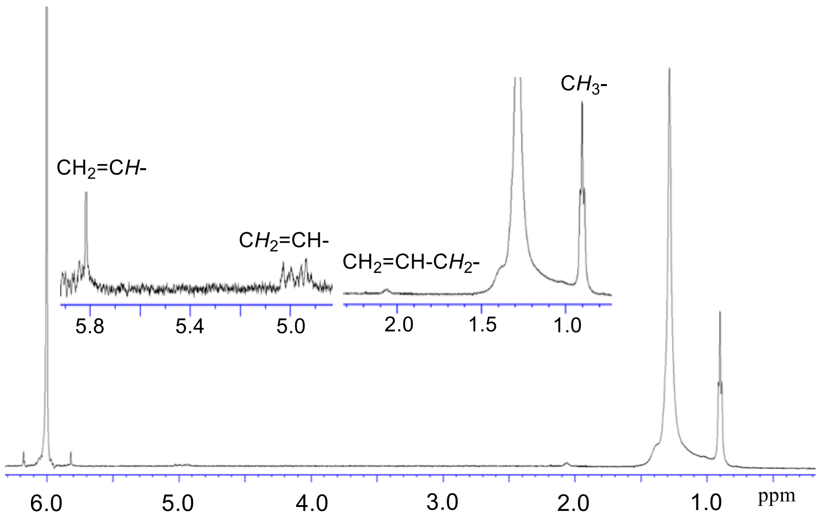

Figure S1. ^{1}H NMR spectrum (in 1,1,2,2-tetrachloroethane-d_2 at 25 ºC) for poly(1-decene-*co*-1,9-decadiene) (run 1, after 5 min, 1,9-decadiene 8.9 mol%).

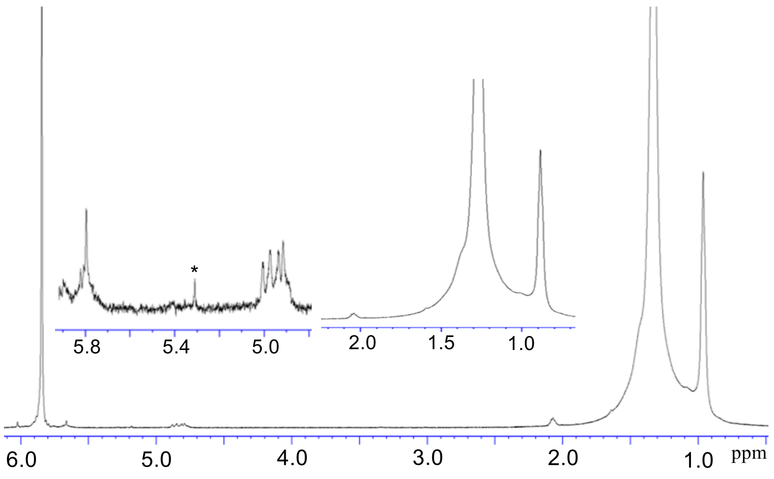

Figure S2. ^{1}H NMR spectrum (in 1,1,2,2-tetrachloroethane-d_2 at 25 ºC) for poly(1-decene-*co*-1,9-decadiene) (run 2, after 10 min, 1,9-decadiene 9.1 mol%). *Impurity

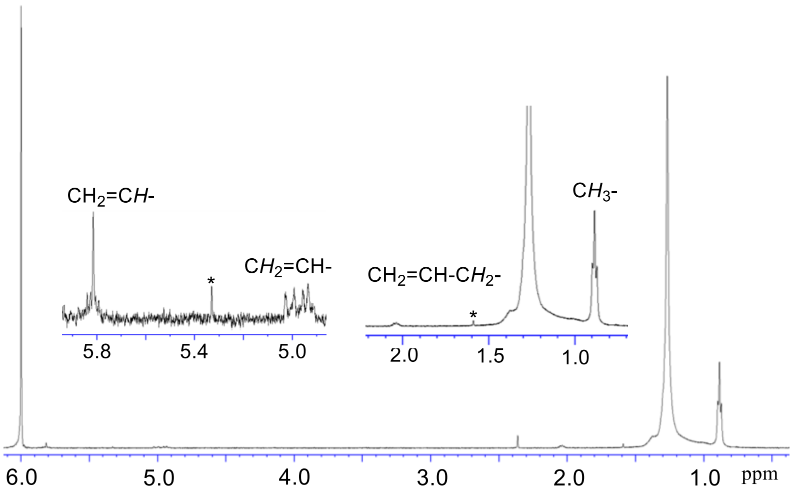

Figure S3. ^{1}H NMR spectrum (in 1,1,2,2-tetrachloroethane-d_2 at 25 ºC) for poly(1-dodecene-*co*-1,11-dodecadiene) (run 4, after 30 min, 1,11-dodecadiene 7.7 mol%). *Impurity

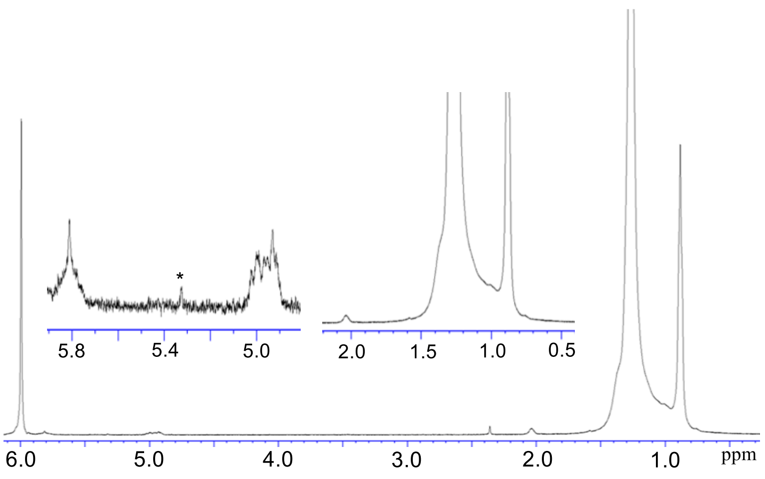

Figure S4. ^{1}H NMR spectrum (in 1,1,2,2-tetrachloroethane-d_2 at 25 ºC) for poly(1-dodecene-*co*-1,11-dodecadiene) (run 5, after 120 min, 1,11-dodecadiene 7.5 mol%). *Impurity

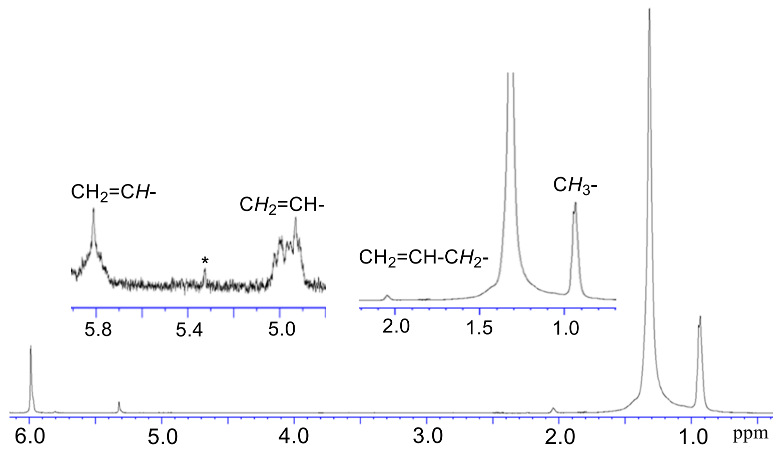

Figure S5. ^{1}H NMR spectrum (in 1,1,2,2-tetrachloroethane-d_2 at 25 ºC) for poly(1-tetradecene-*co*-1,13-tetradecadiene) (run 6, after 30 min, 1,13-tetradecadiene 4.5 mol%). *Impurity

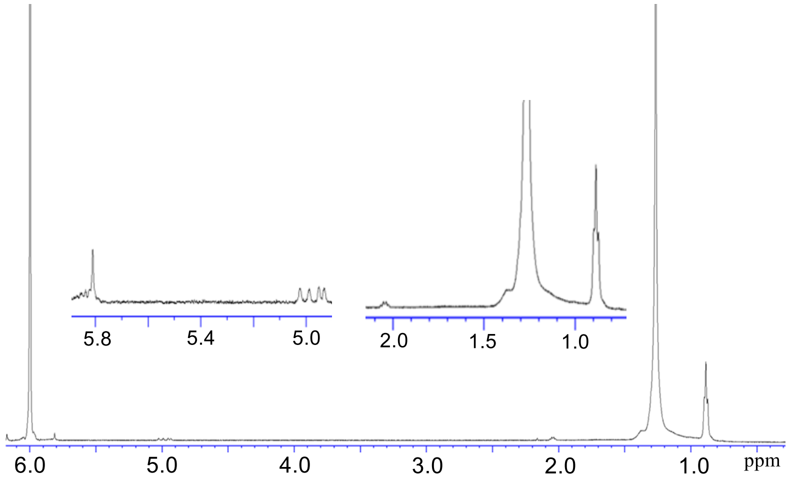

Figure S6. ^{1}H NMR spectrum (in 1,1,2,2-tetrachloroethane-d_2 at 25 ºC) for poly(1-tetradecene-*co*-1,13-tetradecadiene) (run 6, after 60 min, 1,13-tetradecadiene 3.7 mol%).
